# Retention of beneficial molecules and coagulation factors during haemodialysis and haemodiafiltration

**DOI:** 10.1038/s41598-019-42783-w

**Published:** 2019-04-23

**Authors:** Manuel Voigt, Michael Gebert, Ulrike Haug, Michael Hulko, Markus Storr, Adriana Boschetti-de-Fierro, Werner Beck, Bernd Krause

**Affiliations:** Baxter International Inc., Research & Development, Holger-Crafoord-Str. 26, 72379 Hechingen, Germany

**Keywords:** Nephrology, Renal replacement therapy

## Abstract

Middle molecules (MMs) are associated with the pathology of uraemia, and are not effectively removed by standard extracorporeal treatments. Increased convection used in haemodiafiltration (HDF) can enhance the removal of MMs; however, high-volume HDF is not available to all patients. The new medium cut-off (MCO) membrane has been developed to allow increased removal of MMs using standard haemodialysis (HD). Improved removal of MMs has been shown with the MCO membrane compared with standard high-flux dialysers, but it is not known whether the increased pore size affects the retention of commonly used medications or that of coagulation factors in dialysis patients. Using an *in vitro* model, the retention of erythropoietin, heparin, insulin, vancomycin and several coagulation factors (Factors II, VII and X, protein C and antithrombin III) was investigated with the MCO membrane dialyser, compared with high-flux dialysers with polysulfone (in HDF) or polyethersulfone membranes (in HD and HDF). The retention of all molecules investigated was comparable between the MCO membrane and the high-flux dialysers. Results from the *in vitro* studies suggest that switching from a high-flux dialyser to the MCO membrane should not require changes to the medication dosing or anti-coagulation protocols of dialysis patients.

## Introduction

Progressive kidney disease induces uraemic syndrome, which is attributed to the gradual retention of various molecules that are normally excreted by the kidney^1,2^. Middle molecules (MMs) are associated with the pathology of uraemia and play a significant role in uraemic toxicity^2–4^. They consist of molecules ranging from 500 Da up to ~60 kDa; larger MMs (>15 kDa) and are not effectively removed by standard extracorporeal treatments^3,4^. Removal can be enhanced by increased convection with haemodiafiltration (HDF) therapy; however, HDF therapy is complex and may not be suitable for, or available to, all patients^5–8^.

To extend the range of toxins that can be removed during haemodialysis (HD), expanded haemodialysis (HDx) therapy has become available, which is currently enabled by the novel medium cut-off MCO (Theranova) membrane^9,10^. The MCO membrane has an open, three-layer structure, with uniform pore size distribution, that can remove larger MMs up to 45 kDa^11–13^. This promotes removal of large toxins while retaining albumin^9,12,14^. In clinical studies, improved removal performance of MCO membranes was demonstrated allowing clearance of a wide range of MMs more effectively than high-flux HD, and performance of these membranes exceeded that of high-volume HDF for larger MMs (up to 60 kDa)^9^.

The new MCO membrane enables the removal of MMs in standard HD, while simultaneously maintaining a low passage of albumin^9^. However, it is not known whether the increased pore size in the MCO membrane affects the retention of commonly used medications, or functional proteins such as coagulation factors. It is important to know whether there are differences in retention compared with high-flux dialyser membranes to determine if changes to dosing schedules may be required. Therefore, a selection of drugs of varying molecular weights, which are commonly prescribed for dialysis patients, have been investigated. Erythropoietin (30 kDa), low-molecular-weight heparin (LMWH; 5 kDa), insulin (6 kDa) and vancomycin (1.5 kDa) were selected as medications of interest^15–20^. Factor II (71.6 kDa), Factor VII (50 kDa), Factor X (58.8 kDa), protein C (62 kDa) and antithrombin III (ATIII; 58 kDa) were investigated as large coagulation factors of interest^21,22^.

In this *in vitro* study, HD- or HDF-treatment conditions were simulated to investigate loss of various medications and functional proteins during dialysis. The goal was to assess the retention of these molecules and proteins with the polyethersulfone (PES)-based MCO membrane dialyser (Theranova) in HD mode compared with two high-flux membrane dialysers in HD and HDF modes: a PES membrane dialyser (Polyflux 210 H) in HD and HDF modes, and a polysulfone (PSu) membrane dialyser (FX CorDiax 800) in HDF mode. To our knowledge, this is the first study to investigate these properties of the MCO membrane.

## Results

### Erythropoietin

**Figure 1:**
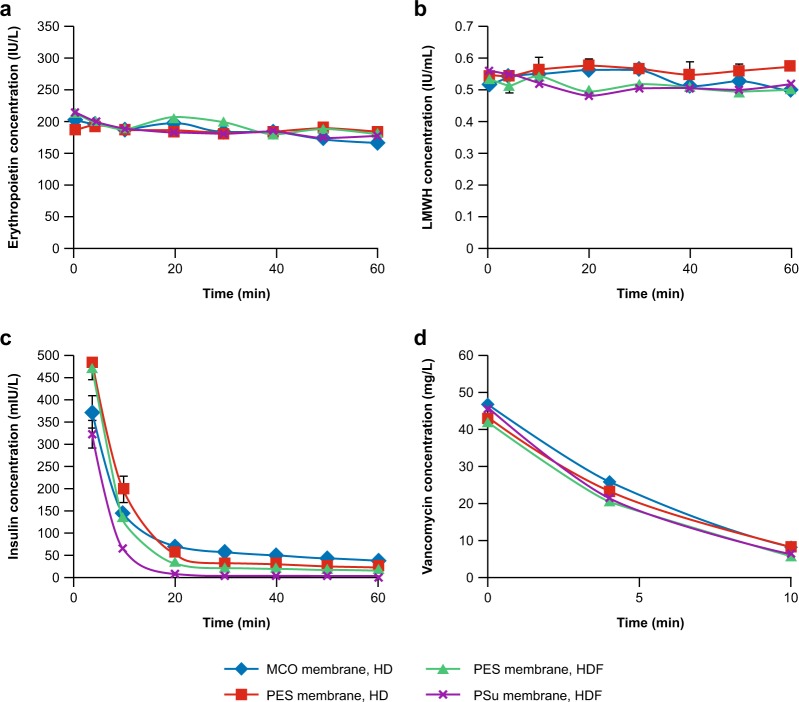
Retention of erythropoietin (**a**), low molecular weight heparin (LMWH) (**b**), insulin (**c**) and vancomycin (**d**) in a simulated treatment with medium cut-off (MCO) and high-flux dialysers. Data are presented as mean (n = 3) ± standard error of the mean (SEM). Insulin concentrations at t0 were out of the range of the insulin assay (>1 IU/L). No consistent starting concentrations could be achieved, and the starting concentration of 1 lU/L was chosen to be high enough so that insulin would still be detectable over the time frame of the experiments. HD, haemodialysis; HDF, haemodiafiltration; PES, polyethersulfone; PSu, polysulfone.

The starting concentration of erythropoietin at time (t) 0 min (t0) was similar for all dialysers tested, with average concentrations of 203, 188, 216 and 214 IU/mL for MCO, PES in HD, PES in HDF and PSu membrane dialysers, respectively. Erythropoietin concentration declined minimally and comparably during simulated treatment with all dialysers in HD and HDF treatment modes (Fig. 1a), remaining above 160 IU/mL at t60 for all membranes tested (165, 183, 182 and 177 IU/mL for MCO in HD, PES in HD, PES in HDF and PSu in HD, respectively). Specifically, the change of erythropoietin concentration observed for the MCO membrane in HD mode was similar to that of the PSu membrane in simulated HDF mode.

### LMWH

Minimal decline in LMWH plasma concentration was observed for all dialysers tested, with the concentration at t60 close to the initial dose of 0.6 IU/mL (Fig. 1b). At t60, the average concentrations were 0.5, 0.57, 0.51 and 0.52 IU/mL for MCO, PES in HD, PES in HDF and PSu membrane dialysers, respectively. LMWH concentrations were comparable for all membranes.

### Insulin

A starting concentration of 1000 mIU/L was targeted; this was considered sufficiently high for insulin to be detectable over the time frame of the experiments. No consistent starting concentrations (t0) could be achieved; insulin levels decreased rapidly for all dialysers and conditions tested, and the lowest levels were observed with the PSu membrane dialyser (Fig. 1c). At t4, the plasma insulin concentration for the MCO membrane dialyser was 373 mIU/L in simulated HD mode with ultrafiltration rate = 0, compared with 474 mIU/L with the PES membrane dialyser (HDF with an ultrafiltration rate of 100 mL/min), and 322 mIU/L with the PSu membrane dialyser in simulated HDF mode. At t60, almost all insulin had been removed from the plasma with the PSu membrane (1.6 mIU/L), but low levels remained with the other dialysers, including the MCO membrane dialyser (up to 38 mIU/L).

### Vancomycin

Vancomycin was cleared from the 1 L plasma pool within 10 min by all dialysers. At t10, average concentrations were 7.1, 8.3, 5.7 and 6.4 mg/L for MCO, PES in HD, PES in HDF and PSu membrane dialysers, respectively. At t10, the concentration of vancomycin was below the detection limit of the assay (<2.5 mg/L). No difference was observed between the MCO membrane dialyser, and the other dialysers investigated (Fig. 1d). Vancomycin clearance was comparable for all membranes (Theranova 500, 182.8 mL/min; FX CorDiax 800, 196.4 ml/min; Polyflux 210 H in HD mode, 162.6 ml/min; Polyflux 210 H in HDF mode, 196 ml/min; R^2^ = 1 for each dialyser).

#### Activity of coagulation factors

**Figure 2:**
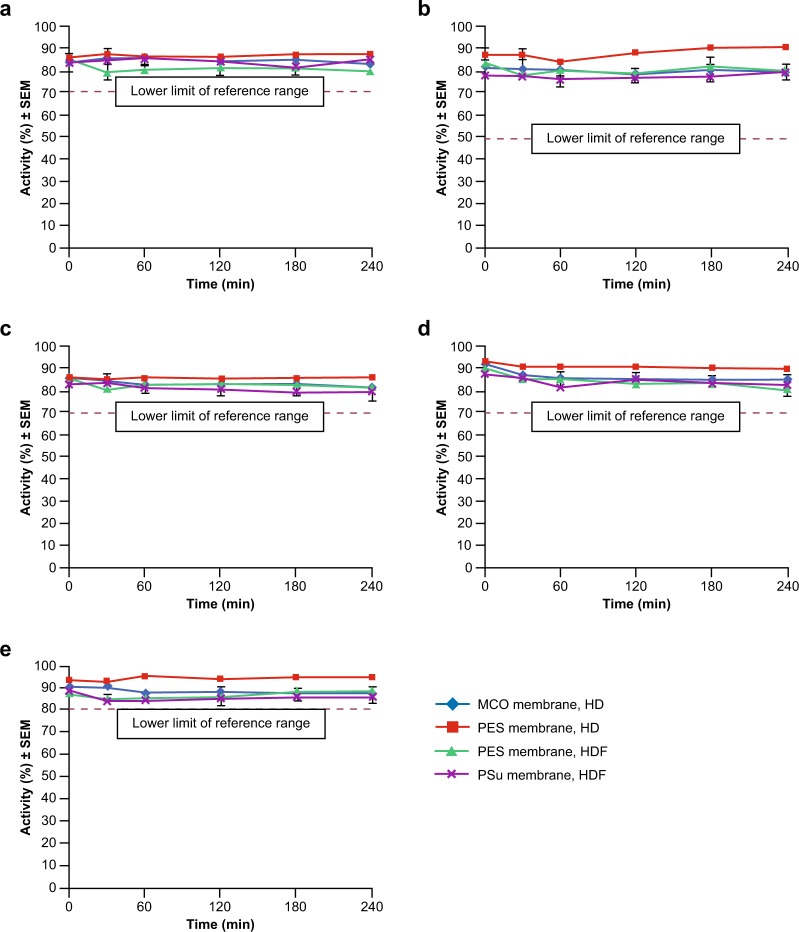
Activity of coagulation factors and inhibitors following treatment with medium cut-off (MCO) and high-flux dialysers towards Factor II (**a**), Factor VII (**b**), Factor X (**c**), protein C (**d**) and antithrombin III (**e**). Data are presented as mean (n = 3) ± standard error of the mean (SEM). HD, haemodialysis; HDF, haemodiafiltration; PES, polyethersulfone; PSu, polysulfone. Normal activity ranges: Factor II 70–120%; Factor VII 50–200%; Factor X 70–150%; Protein C 70–150%; Antithrombin III 80–120%.

The activity of coagulation Factors II, VII and X, protein C and ATIII are shown in Fig. 2. All values measured during the experiments were within the reference ranges for healthy subjects as provided in the assay instructions. Observed mean changes over 240 min were within a range of −9.3% to +3.3% for absolute values and −8.4% to +2.8% for relative values. The highest preservation of all coagulation factor and inhibitor activities investigated was observed with the high-flux PES membrane in simulated HD mode (86.3–95.0% activity at t240). In simulated HD mode, the reported activities with the MCO membrane were in the same range as the values for PES and PSu membranes in simulated HDF mode at t240 (79.0–89.0%). The activities during simulated HDF with PES and PSu membranes were similar at t0 and t240, with values only slightly below those obtained with the PES membrane in HD mode. Activities ranged from 79.0–89.0% for both PES and PSu dialysers at t240. Albumin concentration in the plasma pool appeared to be well preserved with all dialysers and remained within the normal range (35‒55 g/L).

## Discussion

In the *in vitro* setting, the selectivity of the new MCO membrane for erythropoietin, LMWH, insulin and vancomycin was comparable to that of the well-established high-flux dialysers with PES and PSu membranes. Anaemia (a complication of kidney failure), diabetes (frequently HD-induced hypoglycaemia) and other comorbidities are common among patients undergoing dialysis. Therefore, it is important to establish that the expanded removal offered by the new MCO membrane does not negatively impact the various medications required. As such, our data have implications for the use of the new MCO membrane, in addition to PES and PSu membranes, for the management of different dialysis-related complications in clinical practice.

At the terminal stage of chronic kidney disease, patients commonly encounter complex disturbance of the coagulation system leading to considerable morbidity and mortality^16,21–23^. The activity of coagulation factors and inhibitors may be affected by filtration during HD, therefore the activity of selected coagulation factors and inhibitors was investigated to examine whether there was increased loss with MCO HD compared with standard high-flux HD. The molecular weights of the selected proteins, Factors II, VII and X, protein C and ATIII, are in the same region as that of albumin (68 kDa), and therefore these proteins are interesting as markers of the loss of larger molecules with the MCO membrane.

Removal of drug molecules was similar between dialyser membranes, as particularly demonstrated by heparin and erythropoietin for which the levels in the pools remained fairly constant. Except for vancomycin, clearance values with a sufficient regression coefficient could not be calculated.

Despite the benefits of subcutaneous delivery^20^, intravenous erythropoietin is still widely used in many countries including Canada, Germany, members of the Gulf Cooperation Council and the US^24,25^. The maintenance of plasma erythropoietin levels observed in this *in vitro* model is consistent with observations from clinical studies in which dosing of erythropoietin immediately before dialysis did not affect the rate of erythropoietin loss with cuprophan (Travenol 2308), polysulfone (HF80), acrylonitrile-sodium methyl sulfonate copolymer (AN69) or polymethyl-methacrylate (PMMA) membranes^16^. In patients with ESRD, the pharmacokinetic characteristics of intravenous erythropoietin during or after dialysis were studied in 8 patients to determine whether significant loss of drug occurred during HD. The rate of erythropoietin loss was not affected by HD immediately after erythropoietin administration, although there were minimal losses at the termination of the procedure in the dialysis system itself when the dialysis membrane was removed^16^. The similar retention of the MCO membrane for erythropoietin compared with the well-established high-flux dialysers suggests that it should not be necessary to adjust treatment protocols of the molecules investigated if switching from a high-flux PES or PSu dialyser to the MCO dialyser.

Based on the size of the pores of the MCO membrane, and the molecular weight of heparin used (5 kDa), LMWH would be expected to pass through the membrane easily, and a decline in concentration would be expected over time^14^. However, LMWH was not removed from the plasma pool in the present *in vitro* experiment. The observed result (concentrations remaining steady over time) may be explained by heparin binding to other proteins in the plasma (e.g., human ATIII; 58 kDa) to form a complex that exceeds the membrane cut-off  ^26,27^, or possibly by the electrical charge of LMWH causing repulsion by the membrane^18^. The initial binding of heparin to antithrombin is weak, but ATIII then undergoes a conformational change resulting in tight binding of heparin, which is essential for its anticoagulant activity^26^. The current *in vitro* study results support the findings from other studies that have investigated the permeability of PSu membranes to LMWH^15,18^. Klingel *et al*. found that LMWH was not removed by haemofiltration, HDF or high-flux HD, and suggested that the low permeability could be due to its negative electrical charge^18^. Ljungberg *et al*. investigated the efficacy and kinetics of LMWH in HD sessions with either a highly permeable PSu membrane or a conventional cuprophane membrane dialyser. The authors demonstrated that LMWH concentrations in plasma were similar for the two membranes at all given time points^15^.

In the present study, insulin levels decreased rapidly with all dialysers. Of interest, for PES membranes, the insulin level reached a plateau at t20 suggesting that removal by PES membranes may be explained by a simple kinetic model of transport across the membrane. Conversely, insulin was rapidly and completely cleared from the plasma with the PSu membrane dialyser in simulated HDF by t20. With the MCO membrane, insulin levels remained just below 50 mIU/L at t60. Modelling of insulin kinetics is complex^28^. The starting concentration of 1000 mlU/L was chosen so that it could be detectable over the time frame of the experiments.

The mechanism underlying the rapid clearance of insulin seen here is unknown but is unlikely to be related to pore size given the observed kinetics; that is, the removal observed with the MCO membrane was comparable to that seen with other membranes with smaller pore sizes. It could be caused by potential differences in the starting conditions or a second mechanism of removal such as membrane adsorption. Previous *in vitro* data have shown that tubing materials, e.g., polyethylene (PE) and polyurethane (PUR), will adsorb insulin differently. Significantly less insulin was adsorbed by the PUR tubing system compared to the PE tubing system. If PUR tubing is used, less insulin may need to be given to patients for effective blood glucose control^29^. In a study conducted by Quellhorst, insulin requirement per day was determined in 16 HD, 18 haemofiltration, 26 continuous ambulatory peritoneal dialysis (CAPD) and 14 intermittent peritoneal dialysis (IPD) patients at the onset of diabetes, at the start of artificial kidney treatment and 18 months after the beginning of diabetes therapy. In CAPD and IPD patients, insulin requirements were increased when given intraperitoneally^30^. This may be due to the effect of dilution and adsorption and thus inactivation of insulin by plastic surfaces of the tubing and dialysis fluid bag^30^.

Although the half-life of insulin in plasma is short *in vivo* (approximately 4–6 min)^28^, the steep decline in insulin concentrations seen in the current experimental study may be due to a mixture of degradation and removal/adsorption by the dialysis membrane. Although proteolytic degradation of insulin will be an important factor, it is possible that the differences seen here in insulin clearance between the PSu membrane (with all insulin removed) and PES and MCO membrane dialysers may be due to the membrane composition, that is adsorption to the membrane. However, further investigation of this possibility is needed. Our study results agree with the findings of a clinical study in non-diabetic ESRD patients by Jørgensen *et al*. suggesting that insulin was cleared across the membrane via both diffusion and convection using the same PES high-flux dialyser, which may influence the electrochemical and hydrophilic/hydrophobic properties and hence the ability to adsorb insulin. The authors found significantly lower clearance of insulin in simulated HD mode (mean; 95% confidence interval: 84.4; 66.0–102.8 mL/min) compared with HDF mode (mean; 95% confidence interval: 107.6; 87.1–128.0 mL/min)^19^. Regardless of removal mechanism, our data should be interpreted in the context of current recommendations for the administration of insulin to patients with diabetes who require dialysis.

High levels of vancomycin clearance were observed for all membranes investigated in the present study, with no difference seen with the MCO membrane dialyser. High clearance with all dialysers was expected as vancomycin is a relatively small molecule that is almost exclusively cleared by the kidney *in vivo*^31^. Furthermore, levels of protein-bound vancomycin are expected to be low in our model; studies have shown that vancomycin displays moderate protein binding (~40%) but levels are lower in dialysis patients, possibly due to reduced albumin-binding affinity and competition with endogenous substrates that accumulate with reduced renal clearance^32^. Therefore, free vancomycin in the plasma test pool would have the opportunity to clear the membrane irrespective of any (temporary) binding to albumin. Previous studies have also found substantial loss with high-flux dialysers with different membrane compositions, and recommendations for additional dosing following each dialysis session^17,33,34^. Barth and DeVincenzo described their pharmacokinetic experience with 89 courses of therapy using a 20 mg/kg loading dose followed by 500 mg doses after each dialysis treatment, and compared results obtained with 41 courses using single weekly dosing. The results demonstrated significant removal of vancomycin by high-flux HD^33^. Launay-Vacher *et al*. reviewed studies that investigated vancomycin loss with different dialysers and extracorporeal techniques and concluded that vancomycin was effectively removed when HD was performed using a high-flux membrane such as PSu (HD clearance ranged from 76 to 131 mL/min in various studies)^17^. Foote *et al*. evaluated the pharmacokinetics of relatively high-dose vancomycin (single dose of 25 mg/kg infused at 1 g/h) when administered during high-flux HD using a PSu membrane. Dialysis clearance of vancomycin was high, ranging from 96 to 158 mL/min (mean 130.7 ± 30.0 mL/min)^34^.

Comparable coagulation factor and inhibitor activities were observed between the MCO membrane dialyser in HD, high-flux PSu membrane dialyser in HDF, and high-flux PES membrane dialysers in HD and HDF modes. All coagulation factor activities were within the expected normal ranges. Retention of these molecules, demonstrated by their preserved activity, was seen over the duration of the experiment (240 min), for Factors II, VII and X, protein C and ATIII. All values stayed above the lower limit of normal ranges (Factor II, 70–120%; Factor VII, 50–200%; Factor X, 70–150%; protein C, 70–150%; ATIII, 80–120%).

With the exception of the ATIII data, our results are consistent with the findings of both Vaziri *et al*. and Pedrini *et al*. Sampling pre- and post-dialysis, Vaziri *et al*. found normal or increased plasma antigen concentrations of Factors II, VII and X, reduction in coagulant activities of Factors II and X, and reductions in ATIII activity and antigen concentration in patients with ESRD. The reduction in activities of Factors II and X may be due to a low-grade *in vivo* activation and the subsequent inactivation of these factors leading to a decrease in their coagulant activity, or may be due to the presence of uraemic inhibitors^22^. In a prospective crossover study, Pedrini *et al*. used a proteomic approach to assess the removal of proteins and peptides following post-dilution HDF with PSu (Xevonta) and PES (Polyflux) high-flux membranes^7^. Using this approach, Factors II, VII and X and protein C were not identified in the dialysate for either dialyser, although ATIII was found (levels were lower with the PES membrane)^7^. The disparities with regard to ATIII may be due to the different methods used to obtain the data and to the fact that, as observed in our study, no uraemic toxins were found.

The molecular weights of the coagulation factors studied in the present study (58–71.6 kDa) are in the same region as albumin (68 kDa). For all 5 molecules investigated, minimal loss of activity was observed over the course of the experiment, suggesting that the MCO membrane is predominantly not permeable to molecules >58 kDa. These results could provide a broader implication with regards to the retention of other similarly sized larger beneficial proteins.

These results suggest that the increased pore size in the MCO membrane does not increase its permeability to commonly used medications for dialysis patients or coagulation factors with molecular weights that are similar to that of albumin. Further, for smaller molecules such as vancomycin, removal is not increased with the MCO membrane compared to other high-flux membranes as pore size is not a limiting factor for their removal. Results from this *in vitro* study suggest that no changes in drug dosing or anticoagulation protocols should be required when using the MCO membrane dialyser in HD mode compared with using other PSu or PES high-flux dialysers in HD or HDF modes.

## Methods

### Conditions used for simulated treatments

*In vitro* experiments were performed with the AK 200 Ultra S machine and BL 105 tubing sets (Gambro Dialysatoren GmbH, Hechingen, Germany; a subsidiary of Baxter International Inc.). Treatments were simulated with 1 L human plasma for 60 min to assess the clearance of beneficial molecules (n = 3), or with 3.5 L human citrated plasma (octaplas LG [Octapharma Ltd., Manchester, UK], protein concentration 50 g/L) for 240 min to assess the activity of coagulation factors (n = 3). Data are presented from replicates of multiple observations. For coagulation factors, ultrafiltrate was substituted with calcium-free dialysate fluid (post-dilution). For both sets of experiments, the following conditions were used: blood flow rate, Q_B_ = 400 mL/min; dialysate flow rate, Q_D_ = 700 mL/min; ultrafiltration rate, Q_UF_ = 0 mL/min (HD, beneficial molecules); Q_UF_ = 10 mL/min (HD, coagulation factors only); Q_UF_ = 100 mL/min (HDF) at 37 °C.

### Investigated dialysers

The dialysers included in the study were Theranova 500, Polyflux 210 H (Gambro Dialysatoren GmbH, Hechingen, Germany, a subsidiary of Baxter International Inc.) for HD and HDF, and FX CorDiax 800 (Fresenius Medical Care Deutschland, Bad Homburg, Germany) for HDF (Table 1).

**Table 1:** Characteristics of the investigated dialysers^a^.

Dialyser brand name	Membrane	Membrane polymer	Treatment modality	Surface area (m^2^)	Fibre ID/weight (µm) ± 2%	Water permeability (mL/m^2^ * h * mmHg) ± 5%
Theranova 500	MCO	PES/PVP	HD mode	2.0	180/35	636
Polyflux 210 H	High flux	PES/PVP	HD and HDF modes	2.1	215/50	312
FX CorDiax 800	High flux	PSu/PVP	HDF mode	2.0	200/45	276

HD, haemodialysis; HDF, haemodiafiltration; MCO, medium cut-off PES, polyethersulfone; PSu, polysulfone; PVP, polyvinylpyrrolidone.

^a^Surface areas stated are as listed in published data sheets, fibre dimensions measured for 20 fibres in one filter and water permeability (as flow of purified water at a given transmembrane pressure per membrane surface at 37 ± 1 °C) measured for 6 filters.

### Assessment of beneficial molecule removal

In each experiment, the plasma pool was spiked with all drugs, dissolved in infusion solutions, after recirculation for 55 min. The starting concentrations of drugs, calculated from the spiked amounts, were: 200 IU/L erythropoietin (NeoRecormon, Roche), 0.6 IU/mL LMWH (Fragmin P, Pfizer), 1 IU/L insulin (Actrapid, Novo Nordisk) and 50 mg/L vancomycin. Plasma samples were collected at 0, 4, 10, 20, 30, 40 and 60 min, frozen and concentrations were determined by MVZ Labor Dr. Limbach & Kollegen GbR (Heidelberg, Germany). Clearance values were calculated from first-order kinetics of pool concentrations over time only if the regression coefficient was sufficient.

### Assessment of coagulation factor activity

Samples, without added anticoagulant, were collected from the human citrated plasma (octaplasm LG) pool into uncoated tubes at 0, 30, 60, 120, 180 and 240 min, frozen and analysed for activities (%) of coagulation Factors II, VII and X by electromechanical clot detection and of inhibitors ATIII and protein C by colourimetry at BARC Europe NV (Ghent, Belgium). Coagulation factors studied were based on the results of Meijers *et al*.^35^.

## Data Availability

The datasets generated during and/or analysed during the current study are available from the corresponding author on reasonable request.
